# Economic evaluation of pneumococcal conjugate vaccination in The Gambia

**DOI:** 10.1186/1471-2334-10-260

**Published:** 2010-09-03

**Authors:** Sun-Young Kim, Gene Lee, Sue J Goldie

**Affiliations:** 1 Center for Health Decision Science, Department of Health Policy and Management, Harvard School of Public Health, Boston MA, USA; 2 Harvard Initiative for Global Health, Cambridge MA, USA

## Abstract

**Background:**

Gambia is the second GAVI support-eligible country to introduce the 7-valent pneumococcal conjugate vaccine (PCV7), but a country-specific cost-effectiveness analysis of the vaccine is not available. Our objective was to assess the potential impact of PCVs of different valences in The Gambia.

**Methods:**

We synthesized the best available epidemiological and cost data using a state-transition model to simulate the natural histories of various pneumococcal diseases. For the base-case, we estimated incremental cost (in 2005 US dollars) per disability-adjusted life year (DALY) averted under routine vaccination using PCV9 compared to no vaccination. We extended the base-case results for PCV9 to estimate the cost-effectiveness of PCV7, PCV10, and PCV13, each compared to no vaccination. To explore parameter uncertainty, we performed both deterministic and probabilistic sensitivity analyses. We also explored the impact of vaccine efficacy waning, herd immunity, and serotype replacement, as a part of the uncertainty analyses, by assuming alternative scenarios and extrapolating empirical results from different settings.

**Results:**

Assuming 90% coverage, a program using a 9-valent PCV (PCV9) would prevent approximately 630 hospitalizations, 40 deaths, and 1000 DALYs, over the first 5 years of life of a birth cohort. Under base-case assumptions ($3.5 per vaccine), compared to no intervention, a PCV9 vaccination program would cost $670 per DALY averted in The Gambia. The corresponding values for PCV7, PCV10, and PCV13 were $910, $670, and $570 per DALY averted, respectively. Sensitivity analyses that explored the implications of the uncertain key parameters showed that model outcomes were most sensitive to vaccine price per dose, discount rate, case-fatality rate of primary endpoint pneumonia, and vaccine efficacy against primary endpoint pneumonia.

**Conclusions:**

Based on the information available now, infant PCV vaccination would be expected to reduce pneumococcal diseases caused by *S. pneumoniae *in The Gambia. Assuming a cost-effectiveness threshold of three times GDP per capita, all PCVs examined would be cost-effective at the tentative Advance Market Commitment (AMC) price of $3.5 per dose. Because the cost-effectiveness of a PCV program could be affected by potential serotype replacement or herd immunity effects that may not be known until after a large scale introduction, type-specific surveillance and iterative evaluation will be critical.

## Background

Acute respiratory disease (mainly pneumonia) represents the single most significant cause of deaths in children under 5 years of age worldwide, leading to approximately 2 million annual childhood deaths [1]. Most of the disease burden occurs in developing countries [2,3]. While the etiology of pneumonia is diverse, *Streptococcus pneumoniae *(*S. pneumoniae*) has been found to be the dominant cause of pediatric pneumonia [4]. *S. pneumoniae *is also known to be the principal agent in serious childhood diseases such as meningitis and sepsis and in less serious but common clinical syndromes such as otitis media, sinusitis, and arthritis [5,6]. Various types of vaccines have been developed to combat pneumococcal diseases. First licensed in 2000, the 7-valent pneumococcal conjugate vaccine (PCV7), Prevnar^® ^(Wyeth Vaccines), is currently the only pneumococcal conjugate vaccine intended for use in infants and young children [7]. Recent trials using a 9-valent pneumococcal conjugate vaccine (PCV9) (Wyeth Vaccines) in The Gambia [8-10] and in South Africa [11,12] have demonstrated efficacy against pneumonia and invasive pneumococcal diseases in developing country settings. Since then, vaccines of higher valence--a 10-valent vaccine (PCV10), Synflorix^® ^(GlaxoSmithKline) [13] and a 13-valent vaccine (PCV13) (Wyeth Vaccines) [14]--have replaced the PCV9 in the pipeline.

Due to the high burden of childhood pneumococcal diseases in developing countries, there have been global efforts to expand access to pneumococcal vaccines in these countries [15]. The World Health Organization (WHO) considers immunization of young children with pneumococcal vaccines a priority [15]. As of January 2009, 11 countries have been approved for support by the GAVI Alliance for pneumococcal vaccines [16], and, as of January 2010, two of the countries, Rwanda and The Gambia, have introduced the vaccine into their routine infant immunization programs. In addition, the GAVI Alliance has officially initiated its pilot Advance Market Commitment (AMC) project for accelerating pneumococcal vaccine introduction into developing countries [17,18].

The Gambia is one of the lowest-income countries eligible for the GAVI Alliance support (with GDP per capita of $360 [2005 US$] for 2008 [19]). The country has a high level of childhood mortality (114 per 1,000 live births [20]) and a high burden of pneumococcal diseases, with about 15.5% of child deaths attributable to pneumonia [20]. Under the GAVI's current co-financing scheme, The Gambia is classified as a "poorest" country and is required to pay $0.15 per dose of PCV7, which is the 3^rd ^new vaccine introduced into the country under GAVI support [21]. Despite this level of financial support, given that other new vaccine programs (e.g., hepatitis B and *Haemophilus influenzae *type b [Hib]) are competing for financing, The Gambia needs to be conscious of the financial sustainability of its PCV program. Accordingly, it would be crucial for both local and global policy makers to be provided with information on the health and economic impact of any new pneumococcal vaccine introductions in the country.

Only a few studies have evaluated pneumococcal vaccines in low-income countries [22-24]. Sinha and colleagues [24] assessed cost-effectiveness of childhood pneumococcal vaccination in the 72 GAVI-eligible countries, based on efficacy data from the Gambian clinical study [8]. While their study has provided valuable insights into the cost-effectiveness of pneumococcal vaccines in the lowest-income countries, it primarily focused on the impact of PCV9 on overall child mortality, since detailed data on the incidence of other clinical endpoints were not available for all 72 countries [24]. Thus, while their study has projected the potential impact of PCV9 in countries with varying levels of childhood mortality, country-specific results based on detailed local data are not available. Recently, more specific epidemiological data (e.g., age group-specific incidence of pneumonia and local serotype distribution) and cost data (e.g., a long-term cap price for PCV suggested by AMC) have become available. In addition, WHO has recently recommended a standardized method for radiological diagnoses and classification of pneumonia in order to facilitate comparison of the results of vaccine trials and epidemiological studies of pneumonia [25,26]. The new guidelines define the radiological criteria for "primary endpoint pneumonia" (presence of lobar consolidation or pleural effusion) as well as clinical criteria for all pneumonias [25,26]. Our objective was to synthesize the best available data in a decision analytic model following the WHO's recent classification system and to re-assess the impact of PCVs of different valences (PCV7, PCV9, PCV10, and PCV13) in The Gambia.

## Methods

### Analytic overview

We developed a computer-based state-transition model that simulates the natural history of pneumonias (both primary endpoint pneumonia as defined by WHO [25,26] and non-primary endpoint pneumonia) and serious clinical forms of pneumococcal diseases in order to evaluate the cost-effectiveness of routine vaccination with PCVs, compared to no vaccination from the societal perspective. While the currently used PCV7 [27] and its likely replacement (PCV10 or PCV13) [28] are of principal interest in this study, we chose to assume a PCV9 intervention for the base-case analysis and adjusted the base-case vaccine impact estimates for PCV7, PCV10, and PCV13. Our choice of PCV9 for the base-case analysis was primarily motivated by the availability of local clinical trial data for PCV9 [8,9] and comparability with the previous study that evaluated the cost-effectiveness of PCV9 [24]. Health outcomes included the numbers of cases, hospitalizations, and deaths attributable to each of the diseases (both endpoints of pneumonia, pneumococcal meningitis, and pneumococcal sepsis). We translated the estimated health outcomes averted into disability-adjusted life years (DALYs) averted using standardized methods [29], with and without (base-case) age-weighting, as recommended by recent WHO guidelines [30]. From the societal perspective, we included transportation costs and caregivers' time costs in addition to direct medical costs (vaccination program costs and medical treatment costs). The primary outcome measure was presented as the incremental cost (2005 US$) per DALY averted, and both costs and DALYs were discounted at 3% for the base-case. We conducted both deterministic and probabilistic sensitivity analyses to explore parameter uncertainty. We also explored the effects of potential herd immunity, serotype replacement, and vaccine efficacy waning by conducting secondary analyses of different scenarios.

### Model

**Figure 1:**
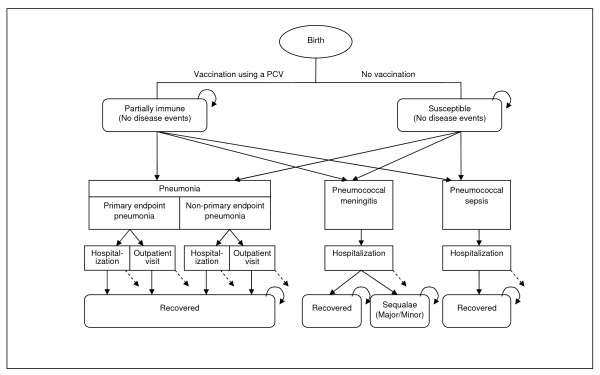
**Model schematic**. This figure presents the schematic of the Markov model. Solid arrows represent transition probabilities between health states, which are differentiated depending on immunization status. Dashed arrows represent disease-specific deaths. Curved arrows represent that individuals stay in the same health states during the next cycle. Deaths due to other causes occur at every stage according to the background mortality rate for Gambian children but are not shown.

The model was structured as a static, aggregate-level, state-transition model with a cycle length of one month (TreeAge Pro 2008). The model simulates the natural history of three different diseases--pneumonia, meningitis, and sepsis--known to be caused by *S. pneumoniae*. For pneumonia, we dichotomized all cases into 'primary endpoint' and 'non-primary endpoint' pneumonias based on the standards recommended by WHO [25,26]. While the present study is concerned with pneumonia due to *S. pneumoniae*, the most comprehensive data available for local incidence of pneumonia measure incidence of all-cause pneumonia, largely since etiology can be confirmed only in very few cases. We therefore chose to use a "vaccine probe" approach in a broad sense [31]; that is, we simulate the occurrence of all-cause pneumonia of both primary and non-primary endpoints for both vaccination and no vaccination strategies, assuming that the net difference in the pneumonia disease burden reflects the burden caused by *all *serotypes of *S. pneumoniae *that is averted under a PCV intervention. For meningitis and sepsis, we modeled disease events by *S. pneumoniae *(all-serotype) only. We applied the model to a hypothetical Gambian birth cohort (N = 60,000) and estimated the clinical and economic consequences of a PCV intervention over the first 5 years of life, based on the following assumptions: (1) repeat infection by *S. pneumoniae *of the same serotype would not occur over the 5-year time horizon (mainly due to insufficient data on partial immunity following natural infection), although diseases may re-occur due to different pneumococcal serotypes or different etiologic agents (e.g., pneumonia due to Hib or meningococcal meningitis); (2) pneumonia might be treated in either inpatient or outpatient settings depending on severity, but, given the general severity of the diseases, all meningitis and sepsis cases would require hospitalization; and (3) a portion of pneumococcal meningitis patients would experience neurological sequelae such as vision loss, hearing loss, motor delay, and seizures upon recovery from the acute illness [32]. Figure 1 shows the schematic of the model.

### Intervention: Vaccine type, coverage, schedule, and efficacy

After conducting a base-case analysis assuming PCV9, we further applied local serotype distribution data to estimate the cost-effectiveness of PCV7, as well as of PCV10 and PCV13. For the base-case, we assumed: (1) a vaccination uptake rate of 90%, which is the coverage rate of a full course of diphtheria-tetanus-pertussis (DTP) and of the two other vaccines (hepatitis B and Hib) recently introduced in The Gambia [33], would be achieved; (2) covered children would receive a full 3 doses of PCV at 2, 4, and 6 months of age; and (3) adverse events from PCVs are negligible, given the demonstrated safety of PCVs [8,34].

The efficacies of PCV9 against primary endpoint and non-primary endpoint pneumonias for children less than 29 months were calculated from the age group-specific incidence data for vaccinated and non-vaccinated children reported in a recent clinical study [9]. The calculated average vaccine efficacies against different endpoints of pneumonia (35% against primary endpoint pneumonia and -1.5% against non-primary endpoint pneumonia) were extrapolated to the age group 30-59 months, assuming the same level of efficacy as the average for children less than 29 months. While this assumption was based on serological response data obtained from a long-term immunogenicity study [11], this assumption was varied in a sensitivity analysis. For pneumococcal meningitis and sepsis, we used the vaccine efficacy against invasive pneumococcal diseases caused by all-serotypes of *S. pneumoniae *(22%), as reported elsewhere by trial investigators [8]. We assumed the same level of efficacy against each type of pneumococcal disease after the first one or two doses of the vaccine as after the full course [35]. Efficacies of PCVs of different valences against each type of pneumococcal disease were estimated by adjusting the base-case (PCV9) vaccine efficacy for the vaccines' serotype coverage and the pathogen's serotype distribution (Table 1). We did not adjust vaccine efficacy for HIV-infected children, since the prevalence of HIV among Gambian children is <1% [19].

**Table 1:** Assumptions on epidemiology, natural history, and disability weights

Parameters	Baseline estimates	Ranges^a^	Distributions^b^	Sources
**Epidemiological parameters**				
Incidence of primary endpoint pneumonia (per 1000/person-year)^c^				
< 6 months	32	25-41^d^	Not varied	[9]
6-11 months	49	42-56^d^	Not varied	[9]
12-17 months	46	40-54^d^	Not varied	[9]
18-23 months	42	36-50^d^	Not varied	[9]
24-29 months	20	15-27^d^	Not varied	[9]
30-59 months	5	3-7	Not varied	[9]
Incidence of non-primary endpoint pneumonia (per 1000 person-year)^c^				
< 6 months	165	144-190	Not varied	[9]
6-11 months	184	166-203	Not varied	[9]
12-17 months	255	229-278	Not varied	[9]
18-23 months	214	193-237	Not varied	[9]
24-29 months	155	135-181	Not varied	[9]
30-59 months	27	23-31	Not varied	[9]
Estimated incidence of pneumococcal meningitis (per 100,000-year)^c^				
< 2 months	12.1	9.7-14.5	Not varied	Estimated
2-11 months	5.2	4.2-6.2	Not varied	Estimated
12-59 months	0.5	0.4-0.6	Not varied	Estimated
Ratio of incidence of childhood meningitis to sepsis attributable to *S. pneumoniae*	2.2	1.0-3.4	Triangular	[5,43]

**Natural history**				
Case-fatality rates				
Primary endpoint pneumonia	3.0%	2-4%	Triangular	[9]
Non-primary endpoint pneumonia	1.1%	0.8-1.4%	Triangular	[9]
Meningitis (age-specific)				
< 1 months	27%	20-34%	Not varied	[41]
1-5 months	23%	17-29%	Not varied	[41]
6-11 months	48%	36-60%	Not varied	[41]
12-59 months	46%	35-58%	Not varied	[41]
Sepsis	35%	26-44%	Triangular	[42]
Proportion hospitalized				
Primary endpoint pneumonia	53.0%	40.0-66.3%	Triangular	[9]
Non-primary endpoint pneumonia	19.3%	14.5-24.1%	Triangular	[9]
Meningitis	100%	70-100%	Uniform	Assumed
Sepsis	100%	70-100%	Uniform	Assumed
Proportion of major disabilities (among disabled due to meningitis)	50%	40-60%	Triangular	[64]

**Vaccine characteristics**				
Vaccine coverage (3 doses)	90	82-100	Triangular	Assumed
Vaccine serotype coverage				
PCV7 (4, 6B, 9V, 14, 18C, 19F, 23F)	46%	Not varied	Not varied	[47]
PCV9 (1, 4, 5, 6B, 9V, 14, 18C, 19F, 23F)	62%	Not varied	Not varied	[47]
PCV10 (1, 4, 5, 6B, 7F, 9V, 14, 18C, 19F, 23F with non-typeable *H. influenzae*)	62%	Not varied	Not varied	[47]
PCV13 (1, 3, 4, 5, 6A, 6B, 7F, 9V, 14, 18C, 19A, 19F, 23F)	73%	Not varied	Not varied	[47]
Vaccine efficacy against (all-cause) primary endpoint pneumonia				
PCV7	26%	20-33%	Triangular	Estimated
PCV9 & 10	35%	26-44%	Triangular	[9]
PCV13	41%	31-51%	Triangular	Estimated
Vaccine efficacy against (all-cause) non-primary endpoint pneumonia				
PCV7	-1.1%	-2.0 to 0%	Triangular	Estimated
PCV9 & 10	-1.5%	-2.0 to -1.0%	Triangular	[9]
PCV13	-1.8%	-2.5 to -2.0%	Triangular	Estimated
Vaccine efficacy against pneumococcal meningitis/sepsis				
PCV7	16%	14-18%	Triangular	Estimated
PCV9 & 10	22%	19-25%	Triangular	[8]
PCV13	26%	22-30%	Triangular	Estimated

**Disability weights**				
Meningitis (aged 0-14), episode	0.616	0.462-0.77	Triangular	[29]
Meningitis, recovered with long-term disability	0.555	0.416-0.694	Triangular	[29]
Pneumonia (aged 0-14), episode	0.28	0.21-0.35	Triangular	[29]
Sepsis, episode	0	Not varied	Not varied	[29]

^a ^This column shows the ranges of parameters that were varied during one-way sensitivity analyses.

^b ^This column shows the distributions assigned to the parameters that were varied during the probabilistic sensitivity analysis. Since data often did not contain enough information from which to estimate distribution parameters, triangular distributions were chosen for a majority of the parameters.

^c ^Annual incidence rates were converted to monthly transition probabilities within the model, assuming an exponential relationship between the cumulative incidence at different time (age) intervals and time (age).

^d ^95% confidence interval.

### Epidemiological data and assumptions

#### Etiology and case-fatality rates

While *S. pneumoniae *and Hib are the two dominant pathogens in bacterial pneumonia, in The Gambia, Hib is considered to be nearly eliminated due to a successful Hib vaccine introduction [36]. Accordingly, we assumed that about 80% of bacterial pneumonia is attributable to infection by *S. pneumoniae*, based on published literature [5,37-39]. This estimated proportion was used in checking the consistency of data from multiple sources and validating model-projected outcomes with pneumococcal disease burden estimates from an external source [40] (data not shown). For bacterial meningitis, we assumed that about 70% of cases are caused by *S. pneumoniae *[5,41]. This estimate was used in estimating the incidence of pneumococcal meningitis from data on the estimated incidence of all-cause meningitis (see also the next subsection "Incidence rates"). The local case-fatality rates (CFR) for each of the three clinical syndromes (pneumonia, meningitis, sepsis) were obtained from published literature (Table 1) [9,41,42].

#### Incidence rates

For vaccinated and non-vaccinated children aged 29 months and under, age-specific monthly incidence rates of primary endpoint versus non-primary endpoint pneumonias were calculated from age-specific annual incidence rates of four endpoints ('primary endpoint pneumonia,' 'other infiltrates/abnormalities' pneumonia, 'pneumonia with no X-ray abnormalities,' and 'no or unreadable radiograph') reported by trial investigators for participants aged 2-29 months (Table 1) [9]. The middle two categories were classified under non-primary endpoint pneumonia, while "no or unreadable radiograph" cases were assigned proportionately so as to avoid possible misrepresentation of the total cases of pneumonia due to choice of classification. The incidence rates of pneumonia in children aged 30-59 months were estimated based on the average incidence rate (32/1000/year) in participants aged 24-59 months, as separately reported by Enwere et al. [9]. Relative incidence between the various pneumonia endpoints was assumed to be consistent with that for younger children.

The age-specific incidence rates of pneumococcal meningitis among non-vaccinated children were estimated by applying the proportion of all-cause meningitis attributable to pneumococcal infection [5,41], CFR of pneumococcal meningitis [41], and age-distribution of meningitis cases [41] to the incidence rates of all-cause meningitis deaths estimated by the 2004 Global Burden of Disease (GBD) study (Table 1) [1]. For pneumococcal sepsis, in the absence of high-quality data, we estimated the average incidence rate of the disease among children less than age 5 by applying the ratio of the incidence of pneumococcal meningitis to that of sepsis observed among young Gambian children, 2.2 [5,43], to the estimated incidence rates of pneumococcal meningitis.

#### Serotype distribution of *S. pneumoniae*

In order to estimate the vaccine serotype coverage by PCV7, PCV9, PCV10, and PCV13, we reviewed data from several clinical trial and observational studies on the serotype distribution of pneumococcal agents in The Gambia [44-47]. We elected to use the serotype distribution reported by Antonio et al. [47], obtained from the PCV9 trial placebo group, mainly for internal consistency. The estimated vaccine serotype coverages were 46%, 62%, 62%, and 73% for PCV7, PCV9, PCV10, and PCV13, respectively. The estimated coverages were used in adjusting vaccine efficacy estimates for each of the multi-valent PCVs. In so doing, we assumed no difference in serotype distribution for children with pneumococcal pneumonia, meningitis, or sepsis, based on published surveillance data [44].

### Cost data and assumptions on resource use

Table 2 shows selected assumptions, plausible ranges, and distribution of cost variables. All costs were expressed in 2005 US dollars by adjusting for inflation using GDP deflators [19] when applicable.

**Table 2:** Assumptions on resource utilization

Parameters^a^	Baseline estimates	Ranges^b^	Distributions^c^	Sources
**Vaccination costs**				
Vaccine price (per dose), $	3.5	0-10	Triangular	[18]
Vaccine wastage rate,%	10	0-20	Triangular	Assumed
Program costs for vaccine delivery^d ^(per dose), $	0.34	0-0.68	Triangular	Estimated

**Disease treatment costs**, $				
*Medical visits*				
Cost per hospital bedday	5.01	2.51-7.52	Triangular	[49]
Cost per outpatient visit	1.31	0.66-1.97	Triangular	[49]
*Diagnostics*				
Pneumonia (inpatient)	4.18	3.14-5.23	Triangular	[50]
Meningitis	1.19	0.89-1.49	Triangular	[50]
Sepsis	4.10	3.08-5.13	Triangular	[50]
*Medication*				
Pneumonia (inpatient)	Age-specific	1.19-3.25	Not varied	Estimated
Pneumonia (outpatient)	Age-specific	0.04-0.08	Not varied	Estimated
Meningitis	Age-specific	4.26-14.62	Not varied	Estimated
Sepsis	Age-specific	6.13-16.68	Not varied	Estimated
**Direct non-medical costs**, $				
Transportation costs (per travel)	0.41	0.31-0.51	Triangular	[56]
Caregiver's time costs^e ^(per disease event)				
Pneumonia, inpatient^f^	2.16	1.08-3.24	Triangular	Estimated
Pneumonia, outpatient^g^	0.21	0.11-0.32	Triangular	Estimated
Meningitis, inpatient^f^	8.64	4.32-12.96	Triangular	Estimated
Sepsis, inpatient^f^	8.64	4.32-12.96	Triangular	Estimated
Wage^h ^(her hour)	0.09	0.05-0.14	Triangular	Estimated

^a ^All cost estimates are expressed in 2005 US dollars.

^b ^The ranges of parameters are for univariate sensitivity analyses and are chosen to be as inclusive as possible based on the literature.

^c ^Because most data sources for costs did not provide enough information, we assumed triangular forms for all cost parameters.

^d ^Delivery costs included all incremental non-recurrent (capital) and recurrent (operational) program costs incurred under the current Gambian immunization system, beyond the purchase cost of the vaccines themselves and vaccine wastage.

^e ^Caregiver's time costs were calculated based on the human capital approach.

^f ^Caregiver's time costs for inpatient care were calculated using the following formula: the estimated average hourly wage ($0.09/hour) × 8 hours/day × average length of stay for each disease event (3 days for pneumonia and 12 days for meningitis and sepsis).

^g ^Caregiver's time costs for outpatient care for pneumonia were calculated using the following formula: the estimated average hourly wage ($0.09/hour) × average time for travel/waiting/treatment at public health facilities in The Gambia (2.3 hours).

^h ^Average hourly wage for The Gambia was estimated based on data from multiple global sources (e.g., World Development Indicators by the World Bank, The CIA Fact Book, and the International Confederation of Free Trade Unions) taking into account the major industries, employment rate by gender, and size of the workforce in The Gambia.

#### Vaccination program costs

Adopting a societal perspective, we included direct medical costs, which consist of vaccination program costs (vaccine costs and all other delivery costs) as well as medical treatment costs, and direct non-medical costs (travel costs and caregivers' time costs). For the base-case unit price, we used the potential long-term cap price of $3.5 suggested by AMC [18], and we assumed a 10% wastage rate for the vaccine. Since we assumed that any PCV would be provided in the form of a pre-filled syringe as is the case with the currently available PCV7, we did not consider injection supply costs. Program costs for delivering PCVs were estimated based on the Gambian Expanded Program of Immunization (EPI) program cost projections, obtained by analyzing the country-specific data from the WHO's cMYP database (Table 2) [48].

#### Medical utilization and treatment costs

The proportions of children treated at different levels of health facilities (i.e., outpatient clinic vs. hospital) were estimated from published literature [9]. Medical treatment costs included costs for bed days (for inpatient care), outpatient consultations (for outpatient care), diagnostics, and medications. Country-specific unit costs per hospital bed day or visit to an outpatient clinic were obtained from the WHO-CHOICE database (Table 2) [49]. We assumed that diagnostics would only be used at higher level health care delivery settings (i.e., tertiary and some secondary level hospitals) in The Gambia, and in the absence of country-specific data, based diagnostic cost estimates on cost data from Kenya [50]. To estimate medication costs, medical practice patterns (e.g., types of antibiotics and dosage) were estimated based on the Gambian government's standard drug use guidelines [51], while the unit prices for relevant antibiotics were obtained from the International Drug Price Indicator Guide (Table 2) [52]. For each component of the medical treatment costs for pneumonia, the final estimate was calculated as a weighted average of the costs at differing levels of medical care delivery. We assumed that about one third of inpatient care would be provided at the tertiary level with the remainder being provided at secondary facilities [53-55]. For outpatient care, we estimated that a majority (~80%) of care would be provided at secondary level facilities, with tertiary and primary facilities providing only a small portion [53-55]. Inpatient care for meningitis and sepsis was assumed to follow the same pattern as that for pneumonia.

#### Direct non-medical costs

Caregivers' time costs were calculated by multiplying the average time for travel, waiting, and treatment for pneumococcal disease at public health facilities by average hourly wage (Table 2). Transportation cost per trip for inpatient or outpatient treatment was estimated assuming an average public transportation fare of 12 Gambian Dalasis, based on a previous Gambian study [56].

### Uncertainty analysis

#### Sensitivity analysis

Given the high level of uncertainty surrounding the burden of pneumococcal diseases and the long-term vaccine impact on the epidemiology of the diseases, we conducted a comprehensive set of uncertainty analyses. To explore parameter uncertainty, we first performed deterministic sensitivity analyses by varying key parameters one at a time over plausible ranges (Tables 1 and 2). We then performed a probabilistic, multivariate sensitivity analysis by assigning distributions to key uncertain parameters (Tables 1 and 2) and performing 10,000 2nd-order Monte Carlo simulations for each PCV. The results were summarized in the form of cost-effectiveness acceptability curves from the societal perspective.

#### Scenario analysis

To explore the impact of potential vaccine efficacy waning, serotype replacement, and herd immunity effects of PCVs, we assessed alternative scenarios in which we varied our base-case assumptions one at a time and then in combinations. To explore the potential indirect vaccine benefits among non-vaccinated populations, we extended the analytic time horizon from 5 years to a lifetime. We approximated the incidence rates of pneumonia among individuals older than 5 years based on an aggregate number of pneumonia cases reported by local health facilities [57] and on the age-distribution of the burden of lower respiratory infections [1]. We then estimated the impact of PCVs, varying key assumptions about the presence of some possible effects of interest (e.g., waning of vaccine-acquired immunity versus no waning and/or presence of herd immunity versus no herd immunity after a single year intervention). For the level of possible herd immunity, we extrapolated data from a published study conducted in a different setting to the Gambian population, as was done by another previously published study [58,59]. For example, based on Whitney et al.'s results [60], which were observed after the first year of PCV7 implementation (coverage unknown) in the U.S., we assumed 32%, 8%, and 18% decrease in incidence of primary endpoint pneumonia and pneumococcal meningitis and sepsis for unvaccinated individuals aged 20-39 years, 40-64 years, and >65 years, respectively. Additionally, given that the burden of disease is highest in older children and adolescents among individuals aged >5 years in The Gambia, we also varied the incidence of the diseases among unvaccinated individuals aged 5-19 years. Further, given the differences in serotype distributions between those observed in The Gambia and in the U.S., we adjusted the level of extrapolated herd immunity for each PCV using the estimated ratios of the age group-specific serotype coverages in The Gambia versus the U.S. [45,60]. (We recognize, however, that this is a somewhat crude adjustment, given that the level of herd immunity effects would not be necessarily linearly related to the level of serotype coverage. Indeed many other factors may influence the presence and scale of herd immunity effects, such as contact patterns among subpopulations.)

## Results

### Base-case results based on PCV9

Table 3 presents the projected health outcomes and cost-effectiveness estimates for PCV9 intervention compared with no vaccination in The Gambia. Vaccinating a cohort of 60,000 infants, assuming 90% coverage, would be expected to prevent about 1400 cases (~28%) of primary endpoint pneumonia attributable to *S. pneumoniae *and avert hospitalization and outpatient visits due to primary endpoint pneumonia by approximately 740 and 660 cases, respectively. The PCV program would also reduce the cases of meningitis and sepsis caused by the pathogen by about 13% compared with no vaccination. Net health outcomes, including a projected increase in non-primary endpoint pneumonia incidence, hospitalizations, and deaths, amounted to approximately 630 hospitalizations, 40 deaths, and 1000 DALYs averted, over a 5-year time horizon.

**Table 3:** Base-case results using a 9-valent pneumococcal conjugate vaccine (PCV9)

Model outcomes	No vaccination	Vaccination (PCV9)	Reduction
Pneumonia (primary endpoint)	5,039	3,635	27.9%
Pneumonia (non-primary endpoint)	25,123	25,758	-2.5%
Pneumonia (all endpoints)	30,161	29,393	2.5%
Meningitis	46	40	13.2%
Sepsis	21	18	13.2%
			
Hospitalization (primary endpoint pneumonia)	2,670	1,926	27.9%
Outpatient visit (primary endpoint pneumonia)	2,368	1,708	27.9%
Hospitalization (all endpoints of pneumonia)	7,519	6,898	8.3%
Hospitalization (all diseases)	7,586	6,956	8.3%
			
Pneumonia (primary endpoint) deaths	151	109	27.9%
Pneumonia (non-primary endpoint) deaths	276	283	-2.5%
Pneumonia (all endpoints) deaths	428	392	8.2%
Meningitis deaths	16	14	14.7%
Sepsis deaths	7	6	13.1%
			

DALYs (K = 0)^a^	182,630	181,630	0.5%
Costs^a ^(2005 US$)	233,100	902,040	--
ICER (2005 US$/DALY averted)	--	670	--
ICER expressed as % per capita GDP^b^		190%	

DALY: disability-adjusted life year, ICER: incremental cost-effectiveness ratio.

^a ^discounted at 3%.

^b ^$360 (in 2005 US$) in The Gambia for 2008.

The average program cost for vaccinating one child with 3 doses of PCV9 would be $1.02. Assuming $3.5 per dose, 90% coverage, and 10% vaccine wastage rate, the vaccination program cost for the birth cohort would be about $685,000. The total costs, which include medical treatments costs due to pneumococcal infection and non-direct medical costs such as travel and time costs of seeking treatment, would be $902,040 with vaccination and $233,100 without vaccination. Combining the primary health and economic outcome measures, the estimated cost of PCV9 vaccination per DALY averted was $670 (Table 3), compared with no vaccination.

### Extended results for PCV7, PCV10, and PCV13

**Figure 2:**
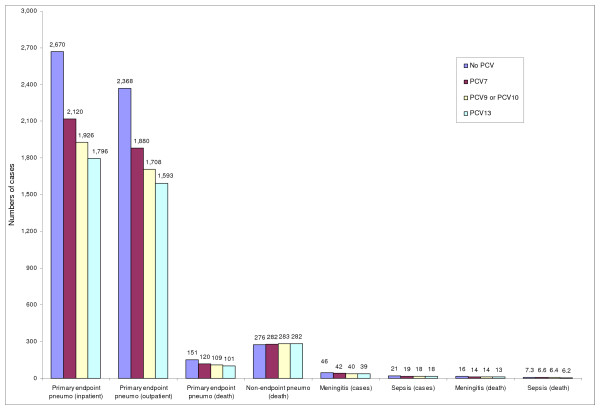
**Selected model-predicted health outcomes**. This figure presents the estimated numbers of cases of different epidemiological outcomes due to *S. pneumoniae *infection in the Gambia according to vaccine type, and compared to no vaccination.

Figure 2 shows the estimated numbers of cases of various epidemiological outcomes by each type of vaccine. It should be noted that the results assuming PCV10 are the same as the ones for PCV9 since the local distribution of 7F, the only serotype differentiating PCV10 from PCV9, was estimated to be zero based on the most recent epidemiological study [47]. Compared to no vaccination, PCV13 would prevent about 1650 cases of primary endpoint pneumonia, while the corresponding figures for PCV10 and PCV7 would be about 1400 and 1040, respectively. Similar patterns were observed in estimating the avertable burden of other types of pneumococcal diseases (Figure 2).

Table 4 summarizes the cost-effectiveness estimates of PCVs of differing valences compared with no vaccination. In the base-case, assuming the same unit price of $3.5 per dose and the same programmatic costs per course regardless of valence, the total costs under PCV7, PCV10, and PCV13 would be $906,240, $902,040, and $899,280, respectively. The differences between the total costs correspond to the differences in direct medical and non-medical costs, which in turn are attributable to different incidence rates of pneumococcal diseases under varying levels of serotype coverage. The numbers of averted DALYs were 740, 1000, and 1180 for PCV7, PCV10, and PCV13, respectively. The incremental cost-effectiveness ratios of the PCVs decreased as the valences of the vaccines increased, with results of $910, $670, and $570 per DALY averted.

**Table 4:** Cost-effectiveness of different types of pneumococcal conjugate vaccines (PCVs)

Outcomes	No vaccination	Vaccination (PCV7)	Vaccination (PCV10)	Vaccination (PCV13)
***K = 0 (base-case)***				
Cost (2005 US$)	233,100	906,240	902,040	899,280
Incremental cost (2005 US$)	-	673,140	668,940	666,180
Effectiveness (DALYs)	182,630	181,890	181,630	181,450
Incremental effectiveness (DALYs averted)	-	740	1,000	1,180
ICER (2005 US$/DALY averted)	-	**910**	**670**	**570**
ICER expressed as % per capita GDP^a^		250%	190%	160%

***K = 1***				
Cost (2005 US$)	233,100	906,240	902,040	899,280
Incremental cost (2005 US$)	-	673,140	668,940	666,180
Effectiveness (DALYs)	208,670	207,820	207,530	207,330
Incremental effectiveness (DALYs averted)	-	850	1,140	1,340
ICER (2005 US$/DALY averted)	-	800	590	500
ICER expressed as % per capita GDP^a^		220%	160%	140%

DALY: disability-adjusted life year, K = 0: uniform age-weights used in DALY calculation, K = 1: non-uniform age-weights used in DALY calculation, ICER: incremental cost-effectiveness ratio.

^a ^$360 (in 2005 US$) in The Gambia for 2008.

### Uncertainty analyses

**Figure 3:**
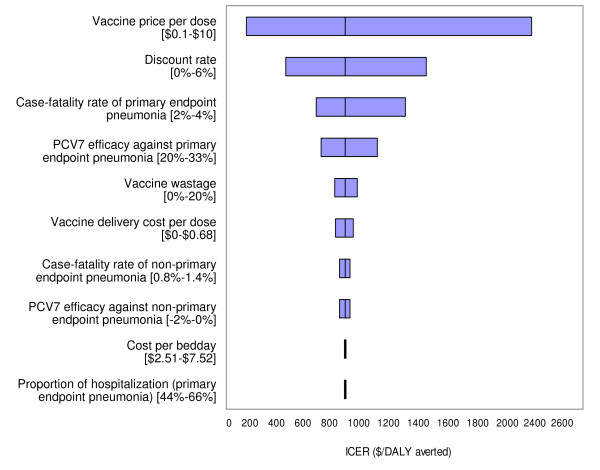
**Results of univariate sensitivity analysis**. The tornadogram shows selected results of univariate sensitivity analysis for PCV9. The x-axis represents the range of the incremental cost-effectiveness ratios for vaccination using PCV7 when the base-case assumptions were varied over plausible ranges (as shown in the brackets). The vertical line represents the base case cost-effectiveness ratio of PCV7, $910 per DALY averted.

**Figure 4:**
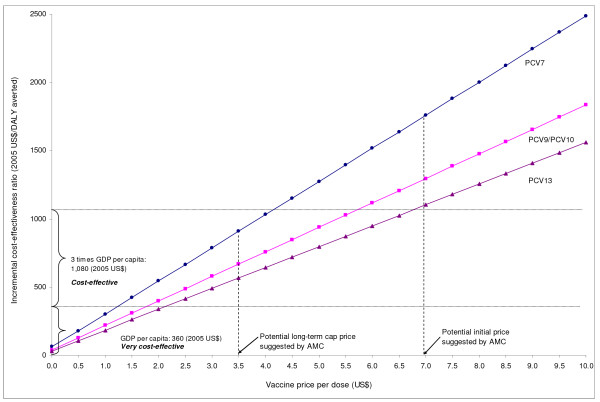
**Deterministic sensitivity analysis: Cost-effectiveness of pneumococcal conjugate vaccines (PCVs) by vaccine price**. This graph shows how cost-effectiveness of each type of PCVs varies as the unit price of vaccines are varied up to $10. The lower horizontal line indicates the threshold cost-effectiveness ratio based on Gambia's GDP per capita. The upper horizontal line indicates three times GDP per capita.

In univariate analyses, results were most sensitive to vaccine price, discount rate, CFR of primary endpoint pneumonia, and vaccine efficacy against primary endpoint pneumonia. Results were moderately sensitive to vaccine wastage rates and vaccination program cost per dose. Results were robust to diagnostic costs, medication costs, outpatient visit costs, wage rates, and transportation costs. When we assumed that vaccine efficacy would be decreased by 15% over a 5-year time horizon, the incremental costs per DALY averted increased to $940, $690, and $590 for PCV7, PCV9 & 10, and PCV13, respectively. When we assumed a 25% decrease in vaccine efficacy up to age 5, the corresponding values for each PCV further increased to $970, $710, and $600. Figure 3 shows a tornadogram summarizing the results of univariate sensitivity analysis using PCV7. Figure 4 presents how cost-effectiveness of each type of PCV varies as the unit price of vaccines are varied up to $10. Using the threshold cost-effectiveness of GDP per capita ($360 in 2005 US$ for 2008), none of the PCVs would be considered *very cost-effective *at the unit price of $3.5, while all the vaccines would be considered *cost-effective *under the threshold of three times GDP per capita.

**Figure 5:**
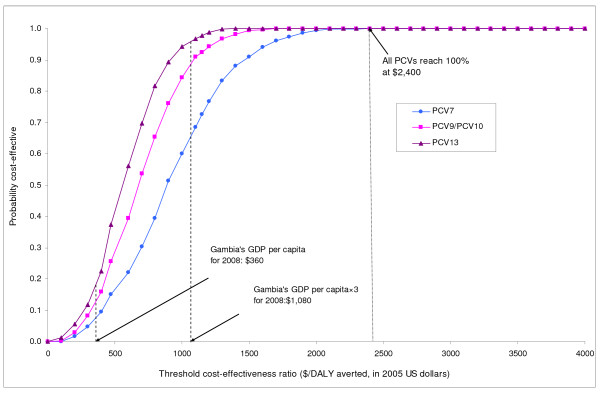
**Probabilistic sensitivity analysis: Cost-effectiveness acceptability curves**. This graph summarizes the results of a probabilistic sensitivity analysis from the societal perspective. The curve shows, for each type of PCVs, the probabilities that pneumococcal vaccination would be cost-effective at varying cost-effectiveness threshold ratios. For example, the probabilities that PCV7 would be cost-effective are 8% and 66% at cost-effectiveness thresholds of $360 (corresponding to The Gambia's GDP per capita) and $1,080 (corresponding to three times The Gambia's GDP per capita) per DALY averted, respectively. All PCVs would be considered 100% cost-effective with the threshold set at $2,400 per DALY averted.

Figure 5 presents the results of a probabilistic sensitivity analysis in the form of a cost-effectiveness acceptability curve for each pneumococcal vaccine. The curve for PCV9 shows that for the base-case the probabilities that the program would be cost-effective are 50%, 75%, and 100% at thresholds of $680, $890, and $1700 per DALY averted. At the threshold of $360, Gambia's GDP per capita, the probabilities that each of the programs would be *very cost-effective *are 8%, 13%, and 18% for PCV7, PCV10, and PCV13, respectively.

Table 5 presents the results of a scenario analysis, expressed in terms of incremental cost-effectiveness ratios under each possible PCV program for selected alternative scenarios (Note that all the scenarios are based on a time horizon extended to lifetime). The results suggest that depending on the combinations of differing assumptions on the possible long-term vaccine effects, the cost-effectiveness profile of each PCV may vary widely. For example, when we assumed that neither vaccine immunity waning nor serotype replacement would occur but that a PCV program would affect carriage status of *S. pneumoniae *among non-vaccinated populations, the incremental cost-effectiveness ratio of PCV7 was $630 per DALY averted. The corresponding value for PCV7 increased to $4110 when we varied the set of assumptions to allow for both immunity waning and serotype replacement but not herd immunity (Table 5). In general, presence of serotype replacement and immunity waning led to higher estimated costs per DALY averted, while assuming herd immunity led to more favorable (lower) incremental cost-effectiveness ratios.

**Table 5:** Results of a scenario analysis

**No**.	Selected alternative scenarios	ICER (US$ per DALY^a ^averted)		
		
		PCV7	PCV9 &10	PCV13
1	Immunity waning (no waning up to age 5, 25% decrease up to age 15, and 50% decrease up to age 30) Serotype replacement (by 25%) No herd immunity	4,110	1,220	1,010

2	No Immunity waning Serotype replacement (by 25%) No herd immunity	3,960	1,170	970

3	No Immunity waning Serotype replacement (increased incidence of non-primary endpoint pneumonia among vaccinated) No herd immunity	900	650	550

4	No Immunity waning No serotype replacement No herd immunity	670	490	410

5	No Immunity waning Serotype replacement (increased incidence of non-primary endpoint pneumonia among vaccinated) Herd immunity (assumed incidence decrease by 32%, 32%, 8%, and 18% for individuals aged 5-19 years, 20-39 years, 40-64 years, and 65 years and older)	830	550	480

6	No Immunity waning No serotype replacement Herd immunity (assumed incidence decrease by 32%, 32%, 8%, and 18% for individuals aged 5-19 years, 20-39 years, 40-64 years, and 65 years and older)	630	430	370

DALY: disability-adjusted life year, ICER: incremental cost-effectiveness ratio.

^a ^DALYs calculated without age-weighting (K = 0).

## Discussion

Our results show that a routine PCV program is expected to prevent, over the first 5 years of life of a birth cohort, approximately 1040-1650 cases of primary endpoint pneumonia, 470-740 hospitalizations due to severe pneumococcal diseases, and 30-50 pneumococcal deaths, as well as avert 740-1180 DALYs, depending on the valences of PCVs (PCV7 to PCV13) used. We found that vaccine price per dose is the main driver of cost-effectiveness, followed by discount rate, case-fatality rate, and vaccine efficacy. Since the UNICEF purchase price of PCV7 or of other vaccines in the pipeline are unknown, in the present analysis we chose to use a base-case value of $3.5, which has been suggested as a possible long term cap price by AMC; we also evaluated the implications of lower and higher costs, including another potential AMC cap price of $7.0 [61]. Using a general heuristic of an incremental cost-effectiveness ratio less than GDP per capita ($360) as a proxy for "good value for money" [62], the results demonstrated that, from the societal perspective, all PCVs examined in the present study-PCV7, PCV9 or PCV10, and PCV13-would be considered *very cost-effective *up to a unit vaccine price of $1.24. Using a higher threshold of three times GDP per capita ($1080), all PCVs were shown to be *cost-effective *up to a per-dose vaccine price of about $4.20.

Note that the cost-effectiveness results for each of the PCVs were calculated using the "no vaccination" baseline comparator. Evaluating each PCV using a comparator of the currently used PCV7 or of the next lower valence of PCV (competing choice analysis) would have been less meaningful to this study, since our analyses do not assume increased programmatic costs or adverse events for higher-valence PCVs. More importantly, using the same baseline comparator of "no vaccination" with which other immunization programs are commonly evaluated allows for comparison not only of current and prospective PCV programs, but also of PCVs against other immunization programs that will ultimately compete for limited financial resources.

Despite the increasing global level efforts to introduce PCVs into developing countries with high burdens of pneumococcal diseases, no published study has provided a detailed assessment on the potential impact of a PCV in an individual, low-income GAVI-eligible country using local data. Our study provides such an assessment for PCVs in The Gambia. In doing so, we took advantage of the availability of recent local data on incidence of pneumonia [9] as categorized by the recent WHO standards [25,26], disease burden of pneumococcal meningitis and sepsis [1,5,41], clinical consequences of pneumococcal diseases [9,63,64], serotype distribution [47], and medical and non-medical costs. Also, our study extends the base-case results for PCV9 to project the effect of PCVs of different valences--PCV7, PCV10, and PCV13. Our findings provide more detailed information on the distribution of the health outcomes across different types of pneumococcal diseases as well as project the potential impact of PCVs based on comprehensive sensitivity analyses that take into account possible waning of vaccine immunity, serotype replacement, and herd immunity.

The results of this analysis are likely a conservative estimate of the health benefits of PCV intervention for the following five main reasons:

• First, we assessed only three main types of pneumococcal diseases, excluding the potential role of PCVs in the prevention of otitis media and serious clinical syndromes, such as cellulitis or septic arthritis, that have also been attributed to *S. pneumonia *infection but for which local data are lacking. Although the burden of these clinical syndromes is relatively small in The Gambia (e.g., according to the 2004 GBD study [1], the annual DALYs for otitis media for the entire population was <500, in contrast to ~58,640 for lower respiratory disease), exclusion of such syndromes underestimates the burden of pneumococcal diseases.

• Second, we assessed the potential impact of PCVs on childhood mortality by simulating vaccine effects on disease-specific mortality. While a 16% reduction in *all-cause *mortality by PCV9 was reported separately by Cutts et al. [8], we relied on incidence, vaccine efficacy, and case-fatality data related specifically to the three major pneumococcal syndromes, as discussed previously. However, given the plausible "hypothesis that pneumonia contributes to many more deaths than are directly attributed to pneumonia in studies using verbal autopsies, and that community-acquired bacteraemia is a greater cause of childhood mortality than previously recognized" [8], our base-case estimates of vaccine effects may be an underestimate.

• Third, we follow the recent WHO standards [25,26] in distinguishing all-cause pneumonias, and assume 35% efficacy against primary endpoint pneumonia while also assuming a 1.5% *increase *in non-primary endpoint pneumonia, based on our interpretation of trial results [9]. Although a negative efficacy value of -1.5% against the latter category may seem small, because more than 80% of estimated cases fall within the category of non-primary endpoint pneumonia, the impact on the aggregate population benefit may be non-negligible. Indeed, in our analysis, the number of deaths due to non-primary endpoint pneumonia is higher among vaccinated than in non-vaccinated children (283 vs. 276), offsetting the number of primary endpoint pneumonia deaths averted by PCV9, 42, by about 17%. Our approach that explicitly considers the impact of PCV on non-primary endpoint pneumonias therefore provides a more conservative assessment of vaccine benefits than most previous discussions, which have focused solely on the reduction in primary endpoint pneumonia.

• Fourth, our base-case analysis conservatively assumed the reported value of 22% for PCV9's efficacy against all-serotype pneumococcal meningitis and sepsis [8], rather than using the theoretical efficacy, for example, of 57% for PCV9, which is computed by multiplying the serotype coverage of PCV9 (62%) by the vaccine efficacy against cases caused by vaccine serotypes (92%) [8].

• Finally, our model does not capture explicitly any herd immunity effects over time among non-vaccinated populations. Given that routine immunization programs using PCV7 have been shown to reduce overall pneumococcal carriage rates in children as well as reduce the incidence of invasive pneumococcal disease in adults through indirect effects [65,66], we may have underestimated the impact of a PCV program.

The weaknesses and limitations of our study relate to both data gaps and a model structure that could not capture indirect effects. Country-specific data were not available for every parameter, and data quality was variable. While our scenario analysis showed that indirect net benefits could lead to more attractive cost-effectiveness ratios, our intention was to gain insight into possible outcomes rather than generate a precise estimate. Caution is needed in interpreting such results due to multiple factors that limit extrapolation of results obtained in developed country settings to developing countries (e.g., differences in immunization strategy, structure and mixing patterns of the population, overall coverage achieved in the population, serotype distribution, and co-morbidities, etc.) [67].

We compared our model-predicted outcomes of hospitalizations and deaths with those from Cutts et al. [8]. While Cutts et al. has reported a 15% reduction (per-protocol analysis) in "first admission" due to all clinical conditions among children receiving PCV9 [8], our base-case analysis predicted an 8% reduction in all hospitalizations. When we adjusted the base-case coverage to 100%, model-predicted reduction in hospitalizations increased to 9%. The remaining difference would presumably be due largely to the fact that Cutts et al. reports only first admissions [8] while our model captures all hospitalization events (including multiple hospitalizations due to repeat episodes by different serotypes) using incidence data from Enwere et al. [9]. Obviously, under this condition, differences between vaccinated and non-vaccinated groups would be less pronounced. Regarding mortality, as previously discussed, while Cutts et al. has reported a reduced overall mortality by 16% among vaccinated children [8], our analysis relied exclusively on disease-specific data and did not incorporate reported vaccine effects on overall mortality. As a result, while our model predicted a 9% reduction in disease-specific mortality under a PCV9 intervention, model-predicted reduction in overall mortality over a 5-year horizon was not remarkable ( < 1%). This suggests that the mortality results between the two studies are not directly comparable, primarily due to the difference in choice of mortality endpoints.

While Sinha et al.'s study [24] does not report country-specific results, according to the under 5 mortality strata of The Gambia, the incremental cost-effectiveness ratio of PCV9 for the country falls between $69 and $138 per DALY averted (in 2000 international dollars). This is based on the study's setting-specific secondary analysis, in which vaccine intervention (at $5 per dose) was credited for reducing hospitalizations and outpatient visits for non-fatal pneumococcal disease cases [24]. The corresponding value from our base-case analysis, $670 per DALY averted (in 2005 US$), is approximately 5 to 10 times higher than that from the previous study. Although there are multiple possible sources of this observed discrepancy (e.g., differences in type and year of currency, model structure, and modeling process), one main source is the fact that Sinha et al.'s health outcome measurement is based on the vaccine's effect on all-cause mortality (i.e., an absolute reduction of 7.4 deaths averted per 1000 vaccinated children) [8] while our study's DALY measurement is based on the vaccine's effect on disease-specific morbidity and mortality (i.e., reduction in disease incidence, hospitalizations, outpatient visits, and deaths due to pneumococcal diseases). Indeed, when we transposed into our model the assumptions made about reduction in all-cause mortality by Sinha et al. and further standardized on some modeling processes (e.g., age-weighting in DALY calculation) and vaccine price, the incremental cost per DALY averted decreased from $670 to $40; this implies that analytic choices about endpoints and assumptions about key parameter values primarily account for the observed differences.

Although our analysis suggests PCVs would be considered a cost-effective investment over a plausible range of future vaccine prices, the base-case estimates of the incremental cost-effectiveness ratios ($570, $670, and $910 per DALY averted for PCV7, PCV10, and PCV13, respectively) are higher than those of other new vaccines that have been introduced in African countries. For example, the costs per DALY averted estimated for a routine hepatitis B vaccination program (with per dose vaccine price of $0.32) in The Gambia was $28 (in 2002 US$) [68], and the corresponding value for the Hib program in Kenya (using a pentavalent diphtheria-tetanus-pertussis-hep B-Hib vaccine at a net cost of $2.46 per dose) was $38 (in 2004 US$) [69]. One of the main drivers of these differences is vaccine price per dose or unit cost of an intervention. For example, when we used the same vaccine price as that for hepatitis B, $0.32, a PCV program's costs per DALY averted decreased to about $80-$140. To a lesser degree, differences in disease burden account for differences in outcomes.

Our study provides insights into the research priorities for accelerating control of pneumococcal disease burden using vaccines. First, our study highlights the importance of future clinical and microbiological studies on the natural history of *S. pneumoniae *and etiology of diseases that are caused by the pathogen. It is well discussed that the burden of pneumococcal diseases is often underestimated even in large-scale clinical trials [9] due to the challenges in isolating the pathogen. Also, little is specifically known about whether natural infection affords any significant protection against reinfection. Once more relevant evidence becomes available through future studies, the burden of pneumococcal diseases might be estimated more accurately. Second, our study also highlights the necessity of more in-depth research on the efficacy of PCVs. As noted previously, a comparison between our study and a previous study [24] shows that the cost-effectiveness profile of a PCV can be highly sensitive to choice of endpoint against which vaccine efficacy is measured. Accordingly, while some hypotheses are available regarding the mechanism by which reduction in all-cause child mortality would be associated with PCV9 intervention [8], more investigation about the impact of PCVs on the all-cause child mortality would be crucial. Third, for any given endpoint, the serotype distribution of *S. pneumoniae*, vaccine serotype-specific efficacy, and possible cross protection against vaccine-related serotypes would be key determinants of PCVs' overall efficacy. As described previously, we observed a large discrepancy between the reported efficacy of PCV9 against, for example, all-serotype pneumococcal meningitis and sepsis (22%) and the theoretical efficacy (~57%), which is calculated as the product of serotype coverage of PCV9 and the vaccine efficacy against invasive diseases caused by vaccine serotypes. Additionally, while our use of surveillance data indicating no role of serotype 7F in local pneumococcal diseases [47] resulted in identical model outcomes for PCV9 and PCV10, other local surveillance data have indicated some role by serogroup 7 (including serotype 7F, which is included in PCV10 and PCV13) [46]. Given that vaccine efficacy is one of the most influential parameters, future clinical studies on this particular subject is warranted. Fourth, on a related note, it is crucial to monitor any trends in potential serotype replacement following large-scale introduction of a PCV into a local setting, since replacement disease would potentially diminish the long-term effectiveness of an immunization program. While our base-case analysis did not consider serotype replacement explicitly, our scenario analysis results show that even a moderate level (e.g., 25%) of serotype replacement could have a large impact on the cost-effectiveness of PCVs in The Gambia. Furthermore, routine immunization of Dutch infants using PCV7 has recently been found not to be cost-effective, due largely to the impact of serotype replacement [70]. Recent surveillance data from different regions have reported an increased incidence of invasive pneumococcal diseases due to non-vaccine serotypes among young children, following the introduction of PCV7 [71-73]. Though confounding factors may contribute to such findings, such findings still highlight the importance of further surveillance and research regarding the presence, magnitude, and speed of replacement by specific serotypes. Lastly, our findings from the scenario analyses suggest that an effective surveillance system should also carefully monitor actual take-up rates of PCVs and post-immunization incidence rates of pneumococcal diseases in order to capture potential indirect effects.

Although The Gambia would receive financial support for a PCV program from the GAVI Alliance according to the co-financing scheme, the country's healthcare expenditures (~$15 per capita per annum [19]) must also accommodate the costs for the two new vaccines (Hepatitis B and Hib) already introduced as well as the six traditional EPI vaccines. Further, the country is also considering introducing another new vaccine, a meningococcal group A conjugate vaccine [74]. Accordingly, in order to provide an optimized set of immunization services in the context of the entire health care infrastructure in The Gambia, it is crucial to assess affordability as well as cost-effectiveness (i.e., value for money) of a PCV program. Technically, affordability of a program can be defined according to whether a fixed (single or shared) budget for a specified period for the program can accommodate the total financial (not economic) costs required to implement the specific program [68,75]. Thus, an accurate and relatively precise estimation of the total financial costs of introducing and sustaining a PCV program from the perspective of a program provider (e.g., local government) as well as an assessment of plausible budget levels would be essential steps for an affordability analysis. A few previous studies illustrate how information on both affordability and cost-effectiveness of a health program can be visually presented, under a single fixed budget [68,75]. If a fixed budget designated exclusively for a PCV program is not available, an affordability analysis for PCV should take a more comprehensive approach, with consideration for other vaccination programs' affordability and shared budget constraints. Also, other complicating issues such as non-additivity of vaccination program costs in the presence of combination vaccines and uncertainty surrounding financial costs should be considered [68,75,76].

## Conclusions

Based on the information available now, infant PCV vaccination would be expected to reduce pneumococcal diseases caused by *S. pneumoniae *and, based on what we know now, is likely to be a cost-effective public health intervention in The Gambia. However, because the cost-effectiveness of a PCV program could be affected by potential serotype replacement or herd immunity effects that may be realized after a large-scale introduction, the importance of iterative evaluation as surveillance data become available cannot be overstated. In addition, the availability, timing of launch, and price of new vaccines with higher valences also is likely to influence both the cost-effectiveness and affordability of PCVs in The Gambia. Accordingly, the health, economic, and financial impact of the pneumococcal vaccine should be re-evaluated as new data become available. Ideally this re-evaluation would be performed in the context of the national immunization program, and formally consider other relatively new (e.g., rotavirus and HPV vaccines) vaccines that may compete for a limited, shared budget in The Gambia.

## Competing interests

The authors declare that they have no competing interests.

## Authors' contributions

SK and SG designed the study. SK and GL performed the literature review and collected data. SK and GL analyzed the data and SK performed the simulations. SK, GL, and SG interpreted the data and results. SK drafted the manuscript with input from SG and GL. SG provided administrative support and technical expertise. All authors reviewed and approved the final version of the manuscript. SK is the guarantor.

## Pre-publication history

The pre-publication history for this paper can be accessed here:

http://www.biomedcentral.com/1471-2334/10/260/prepub
